# Exploring the metabolomic diversity of plant species across spatial (leaf and stem) components and phylogenic groups

**DOI:** 10.1186/s12870-019-2231-y

**Published:** 2020-01-28

**Authors:** Sunmin Lee, Dong-Gu Oh, Digar Singh, Jong Seok Lee, Sarah Lee, Choong Hwan Lee

**Affiliations:** 1 Department of Bioscience and Biotechnology, Konkuk University, Seoul, 143-701 Korea; 2 National Institute of Biological Resources, Environmental Research Complex, Incheon, 22755 Korea; 3 Research Institute for Bioactive-Metabolome Network, Konkuk University, Seoul, 05029 Korea

**Keywords:** Plant parts, Chemodiversity, Antioxidant activity, Tyrosinase inhibition activity, Metabolite profiling

## Abstract

**Background:**

Plants have been used as an important source of indispensable bioactive compounds in various cosmetics, foods, and medicines. However, the subsequent functional annotation of these compounds seems arduous because of the largely uncharacterized, vast metabolic repertoire of plant species with known biological phenotypes. Hence, a rapid multi-parallel screening and characterization approach is needed for plant functional metabolites.

**Results:**

Fifty-one species representing three plant families, namely Asteraceae, Fabaceae, and Rosaceae, were subjected to metabolite profiling using gas chromatography time-of-flight mass spectrometry (GC-TOF-MS) and ultrahigh-performance liquid chromatography quadrupole orbitrap ion trap tandem mass spectrometry (UHPLC-Q-orbitrap-MS/MS) as well as multivariate analyses. Partial least squares discriminant analysis (PLS-DA) of the metabolite profiling datasets indicated a distinct clustered pattern for 51 species depending on plant parts (leaves and stems) and relative phylogeny. Examination of their relative metabolite contents showed that the extracts from Fabaceae plants were abundant in amino acids, fatty acids, and genistein compounds. However, the extracts from Rosaceae had higher levels of catechin and ellagic acid derivatives, whereas those from Asteraceae were higher in kaempferol derivatives and organic acids. Regardless of the different families, aromatic amino acids, branch chain amino acids, chlorogenic acid, flavonoids, and phenylpropanoids related to the shikimate pathway were abundant in leaves. Alternatively, certain amino acids (proline, lysine, and arginine) as well as fatty acids levels were higher in stem extracts. Further, we investigated the associated phenotypes, i.e., antioxidant activities, affected by the observed spatial (leaves and stem) and intra-family metabolomic disparity in the plant extracts. Pearson’s correlation analysis indicated that ellagic acid, mannitol, catechin, epicatechin, and quercetin derivatives were positively correlated with antioxidant phenotypes, whereas eriodictyol was positively correlated with tyrosinase inhibition activity.

**Conclusions:**

This work suggests that metabolite profiling, including multi-parallel approaches and integrated bioassays, may help the expeditious characterization of plant-derived metabolites while simultaneously unraveling their chemodiversity.

## Background

Plants have traditionally been used as an important source of pharmacologically active compounds that maintain human health. Notably, plants have been a major source of numerous antioxidant compounds essential in medicines, cosmetics, and the food industry [1]. In particular, the antioxidant metabolites in fruits and plant extracts have a range of health benefits such as maintaining cardiovascular health and cancer prevention, among many others [2, 3]. In herbal cosmetics, tyrosinase inhibitory compounds from plants are used as anti-melanogenic agents [4]. Tyrosinase, a copper-containing monooxygenase enzyme, is found widely in nature, including in plants, fungi, and animals. It catalyzes the hydroxylation of tyrosine to L-DOPA (L-3,4-dihydroxyphenylalanine), which is subsequently oxidized to L-dopaquinone. This is then auto-polymerized to form melanin pigments [5]. Hence, natural inhibitors of tyrosinase are considered vital for treating dermatological hyper-pigmentation caused by overproduction of melanin [6, 7].

Ushering into the metabolomics era, mass spectrometry (MS)- based metabolite characterization has evolved as an adept methodology to discern chemotaxonomy, metabolic pathways, and phytochemical characterization, complementing the omics-cascade alongside genomics and proteomics [8]. Metabolomics enables unbiased, high-throughput screening and characterization of the metabolite gamut in biological sample extracts through chromatographic separation, high resolution MS, and enhanced detection sensitivity [9–11]. However, the subsequent functional annotation of the identified metabolites often seems difficult, owing to the different titers of metabolic repertoire influencing biological phenotypes. Hence, neoteric multi-parallel approaches need to be explored for the expeditious screening and characterization of functional metabolites in diverse plant samples [12, 13].

It has been observed that plants of the same family usually synthesize compounds of similar classes, owing to the presence of similar biosynthetic pathways and regulatory enzymes [14]. Previously, discriminant metabolites, including the flavonoid contents in plant extracts, have been reported for varying taxonomic orders. However, the spatial metabolic disparity between different plant parts (leaves and stems) has not been deconstructed comprehensively [8, 15]. In general, the chemical composition of different plant parts is largely influenced by genetic factors, nutritional status, and geo-climatic conditions [16]. Moreover, the varying distributions of functional metabolites including leaves, stems, and flowers, necessitating the need to delineate plant chemical diversity across the phylogenies as well spatial components [17–19].

Herein, a multi-parallel metabolomic-cum-bioassay-guided approach toward the metabolomic characterization of different biosystematic groups is proposed for the three different plant families (Asteraceae, Fabaceae, and Rosaceae) across their spatial parts (leaf and stem). We employed untargeted mass spectrometry (MS)-based metabolomics coupled with biochemical phenotype analyses toward the comprehensive characterization of significantly discriminant metabolites contributing to the spatial and phylogenic chemodiversity among different plant species.

## Results

### Metabolite profiling across spatial components (leaves and stems) and families (Asteraceae, Fabaceae, and Rosaceae)

**Fig. 1:**
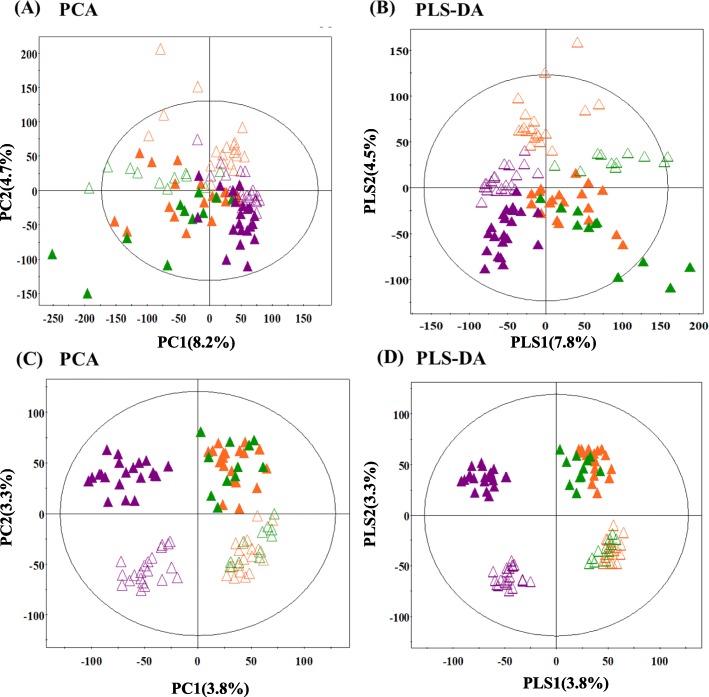
PCA and PLS-DA score plots derived from the (**a**, **b**) GC-TOF-MS dataset and (**c**, **d**) the UHPLC-Q-Orbitrap-MS dataset for leaves and stems of 51 indigenous plant species. (▲; Leaf, △; Stem, Orange, Asteraceae; Green, Fabaceae; violet, Rosaceae)

Herein, we examined the 2-D metabolomic profiles across the spatial components of 51 plant species belonging to three different families (Asteraceae, Fabaceae, and Rosaceae) that are widespread on the Korean peninsula. The subtle metabolic disparity among the plant samples was evaluated using GC-TOF-MS and UHPLC-Q-Orbitrap-MS, followed by multivariate statistical analysis of the corresponding datasets. The principal components analysis (PCA) score plot based on the GC-TOF-MS data displayed a clustered pattern for the 51 samples, segregating them into three distinct groups according to the corresponding plant families across PC 1 (8.2%) and PC 2 (4.7%), while their spatial metabolic disparity was evident along PC 2 (Fig. 1a). Further, the PCA based on the UHPLC-Q-Orbitrap-MS datasets indicated the marked variance across corresponding plant families and plant parts, along PC 1 (3.8%) and PC 2 (3.3%; Fig. 1c), respectively. The significantly discriminant metabolites between the spatial components that potentially contributed to the observed chemotaxonomic variance among the different plant species were statistically selected at variable importance in the projection (VIP) > 0.7 or *p* < 0.05, based on the PLS-DA model (Fig. 1b, d). Altogether, 64 metabolites including 41 primary metabolites and 23 secondary metabolites were identified as significantly discriminant. The primary metabolites were identified using standard compounds, and their spectrometric details are provided as Additional file 1: Table S1. Further, the secondary metabolites were characterized based on their UV-absorbance, masses (m/z), mass fragmentation patterns, and elemental composition (Additional file 2: Table S2). We comprehensively established the putative identities of these compounds using the published methodologies [20]. The metabolites characterized using the above described parameters were comparatively matched with online databases, standards, and *in-house* library, and cross-confirmed using the published literature at last. Intriguingly, the primary metabolite heterogeneity was mainly evident for spatial (leaf and stem) components, while the secondary metabolites were more diversified both spatially and across the plant families.

### Relative metabolite abundance in plant samples across spatial components and families

**Fig. 2:**
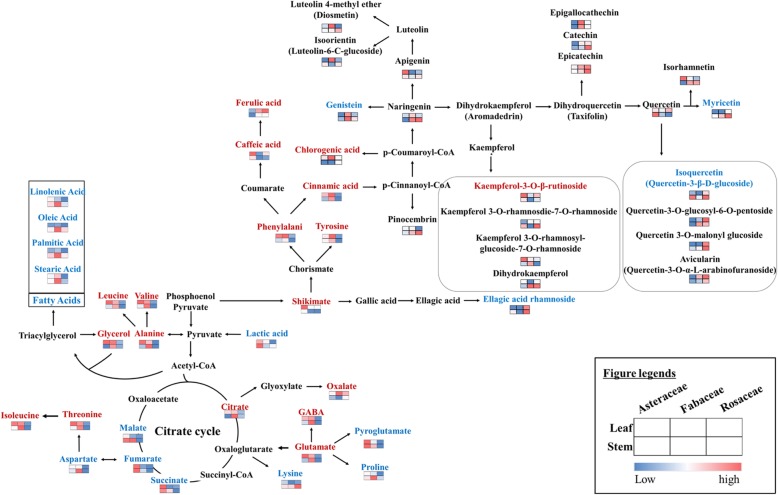
Schematic diagram representing the relative contents of the significantly discriminant metabolites in their corresponding biosynthetic pathways across 51 plant species and across plant spatial parts. The modified pathway was adapted from the KEGG database (http://www.genome.jp/kegg/). The heatmap represents the mean value of the peak area for the discriminant metabolites detected across spatial parts (leaves and stems) for the three plant families (Asteraceae, Fabaceae, and Rosaceae). The discriminant metabolites among leaf and stem extracts are indicated in red and blue fonts, respectively. The colors of the heat map for metabolite levels represent their average fold-change values

The metabolic pathways involved in the biosynthesis of significantly discriminant primary and secondary metabolites were mapped, and their relative abundances in the corresponding plant samples were indicated (Fig. 2). Considering the primary metabolite disparity among the three families, relatively higher levels of organic acids (caffeic acid, lactic acid, succinic acid, shikimic acid, and fumaric acid) were observed in Asteraceae extracts. On the contrary, amino acids (phenylalanine, tyrosine, GABA, serine, arginine, and aspartic acid) and fatty acids (stearic acid, oleic acid, palmitic acid, and oleamide) were abundant in Fabaceae extracts, while some metabolites including adonitol, sorbitol, and ferulic acid were detected at relatively higher levels in Rosaceae family extracts. In the case of secondary metabolites, the relative abundances of kaempferol glucoside and kaempferol-3-O-β-rutinoside were highest in Asteraceae extracts among the three families. On the contrary, genistein, naringenin, and isoorientin were most abundant in Fabaceae, whereas the relative levels of aceroside VIII, isoquercetin, quercetin-3-O-glucosyl-6-O-pentoside, ellagic acid rhamnoside, pinocembrin, epicatechin, and catechin were detected highest in Rosaceae samples.

### Bioactivity correlations for the significantly discriminant metabolites

**Fig. 3:**
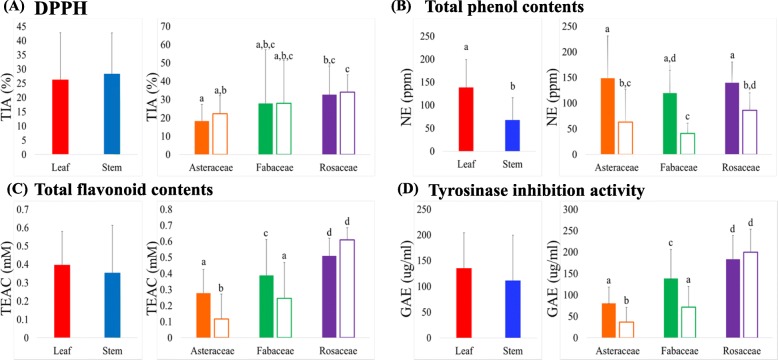
Bioactivities of leaf and stem extracts from 51 indigenous plant species (**a**) Antioxidant activity using DPPH radical scavenging assays, (**b**) total phenol contents, (**c**) total flavonoid contents, and (**d**) tyrosinase inhibition activity

The bioactivity of phytochemical extracts is mainly due to the diverse composition of secondary metabolites fulfilling multiple ecological roles among plant species. The spatial distributions of these compounds among varying phylogenic groups and plant components are remarkably discriminant. In this study, 102 (leaf and stem) sample extracts from 51 plant species were investigated for associated bioactivities using DPPH radical scavenging assays, total phenol content, total flavonoid content, and tyrosinase inhibitory assays (Fig. 3). The results of the bioactivity assays for the 51 species (Family: Asteraceae, Fabaceae, and Rosaceae) were estimated across plant families as well as their spatial components (leaf and stem). The average DPPH antioxidant activity was observed in the following order from highest to lowest; Rosaceae stems > Rosaceae leaves > Fabaceae leaves > Asteraceae leaves > Fabaceae stems > Asteraceae stems (Fig. 3a). Similarly, the average total phenol contents were observed in the following order; Rosaceae stems > Rosaceae leaves > Fabaceae leaves > Asteraceae leaves > Fabaceae stems > Asteraceae stems (Fig. 3b). On the contrary, average values for the total flavonoid content varied in the following order: Asteraceae leaves > Rosaceae leaves > Fabaceae leaves > Rosaceae stems > Asteraceae stems > Fabaceae stems (Fig. 3d). In general, higher antioxidant activity was observed in leaf extracts compared to stem samples, except for the Rosaceae family. On the contrary, average tyrosinase inhibitory activity was significantly higher in stem samples than in leaf samples, irrespective of the plant family.

**Fig. 4:**
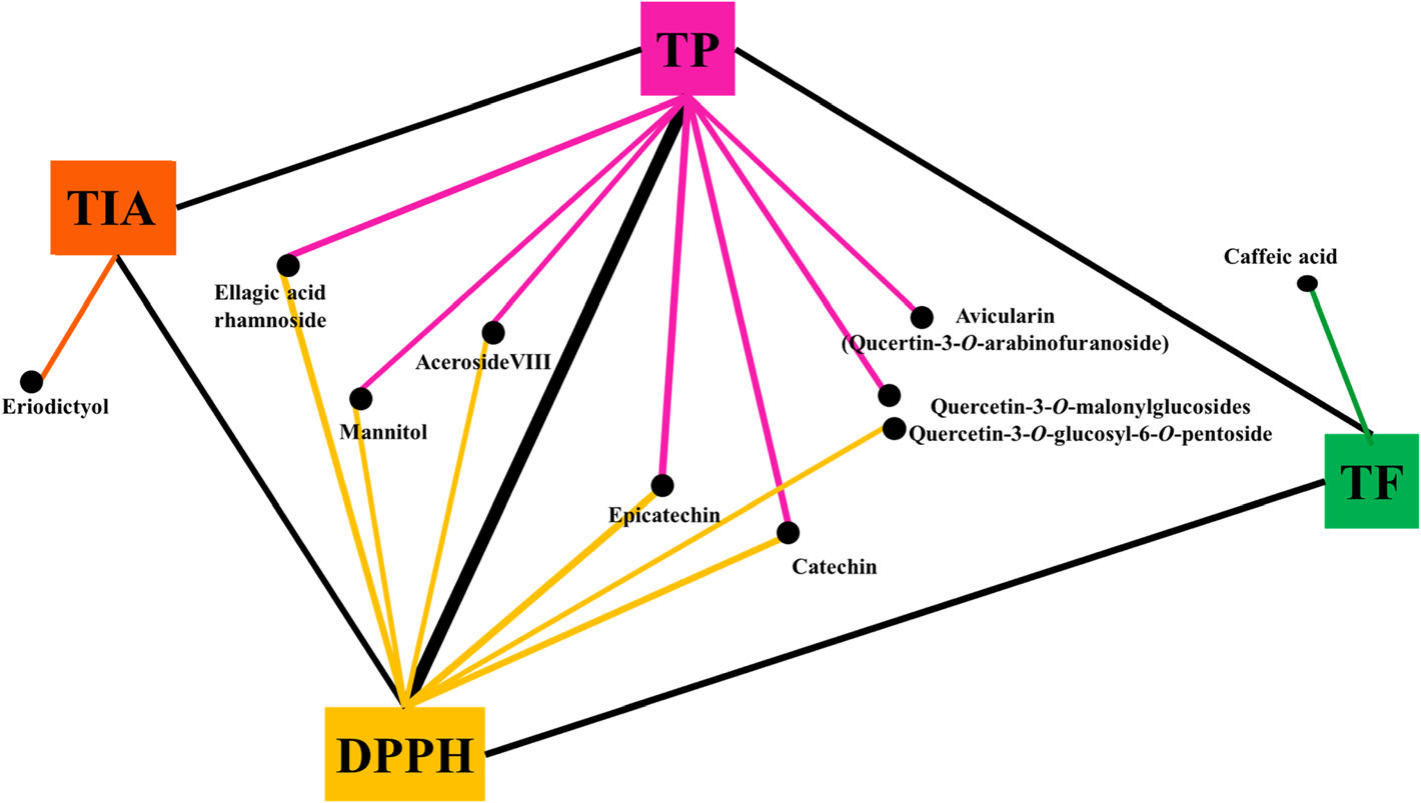
Correlation networks between the metabolites and bioactivity assays (DPPH, TP, TF, and tyrosinase inhibition activity). The metabolites were selected based on a Pearson’s correlation value (r) > 0.3

Pearson’s correlation analysis tentatively identified compounds that contributed maximally to the observed biological activities of the plant extracts. The correlation network was evaluated for variables with a Pearson correlation value > 0.3 (Fig. 4). Intriguingly, aceroside VIII, ellagic acid rhamnose, catechin, epicatechin, mannitol, quercetin-3-O-malonylglucoside, and quercetin-3-O-glucosyl-6-O-pentoside showed strong positive correlations with both total phenol content (TPC) and DPPH antioxidant activity.

## Discussion

We applied untargeted metabolomics coupled with bioactivity assays to evaluate the two dimensional (2D) metabolomic diversity across 51 species belonging to three major plant families and across their spatial plant parts (leaves and stems). The chemical composition of the different plant parts is affected by multiple factors including genetics, metabolic factors, and geo-climatic conditions [14]. Previously, we have highlighted the importance of mass spectrometry (MS)-based metabolomics to identify the chemotaxonomic profiles of different plant samples across various genera and families [8]. The untargeted metabolomics followed by multivariate analyses indicated that metabolite profiles varied significantly for the plant samples across different phylogenies and spatial parts irrespective of the variations in geographical location or in the period of sample collection (2011–2015).

The levels of amino acids are relatively higher in Fabaceae species compared to Cornaceae and Rosaceae [15]. Further, genistein, an important isoflavone is well reported from soybean and other edible Fabaceae species, along with naringenin and isoorientin [21]. In congruence, we observed that amino acids and isoflavones were abundant in Fabaceae, while the kaempferol and quercetin derivatives were relatively higher in Asteraceae and Rosaceae family extracts, respectively. Reportedly, the kaempferol glycosides are the main polyphenolic compounds in Asteraceae family plants [22]. Whereas, quercetin glycosides are the typical flavonol glycoside in the Rosaceae family, along with flavan-3-ol (catechin and epicatechin) and polyphenol compounds, including ellagic acid [23].

Considering the spatial disparity in metabolites between leaf and stem components, the levels of branch chain amino acids (BCAA) (isoleucine, leucine, valine), aromatic amino acids (AAA) (phenylalanine, tyrosine), phenylpropanoids (cinnamic acid, caffeic acid, ferulic acid, shikimic acid), sugar alcohols (xylitol, myo-inositol, meso-erythritol), and glycerol derivatives (glycerol, glyceryl-glyceryl) were relatively higher in leaves than in stem extracts. Branch chain amino acids cannot be synthesized by animals; however, plants can synthesize these amino acids de novo and thus serve as an important source of these compounds in the human diet [24]. In plants, isoleucine, leucine, and valine share common BCAA-hydrolyzing enzymes in their biosynthetic pathways. Accumulation of free amino acids plays an important role in plant stress tolerance, and these can act as osmolytes under certain abiotic stress conditions [25]. Similarly, phenylpropanoids are key components with antioxidant functions that ameliorate high intensity light-stress mediated damage in leaves [26]. On the contrary, the levels of fatty acids (oleic acid, stearic acid, palmitic acid, and linolenic acid) and amino acids (proline, lysine, and arginine) were relatively higher in leaves compared to stem extracts. Oleic and linolenic acid derivatives partially regulate plant development, seed colonization, and defense responses to pathogens through various mechanisms [27, 28]. Notably, the relative abundances of chlorogenic acid and the majority of flavonoids (dihydrokaempferol, quercetin, quercetin derivatives, and myricetin derivatives) were higher in leaves, while kaempferol glucoside and pinocembrin were more abundant in stems. The higher abundance of flavonoids in leaves might be attributed to their local biosynthesis as well as their active translocation from other plant organs at different stages of development [29].

Generally, similar antioxidant activity levels were observed in the same genus groups, but the species belonging to the genera *Alnus* displayed significantly different antioxidant levels. These results suggest that the differences in chemical compositions among species belonging to the same genus may be expressed in terms of their varying chemotaxonomy and associated bioactivities. In the present study, multivariate analyses indicated distinct metabolite profiles for plant extracts according to different plant families and spatial parts. Hence, the chemotaxonomic hierarchy of plants depends on their biosynthetic relatedness to synthesize corresponding metabolite pools [30].

We observed that aceroside VIII, catechin, and quercetin derivatives were positively correlated with DPPH antioxidant phenotypes, whereas eriodictyol was associated with tyrosinase inhibition effects. According to recent studies, aceroside VIII is an acerogenin derivative with significant antioxidant activities [31]. Ellagic acid is structurally a phenol antioxidant that exhibits significant free radical scavenging activity. It also promotes the activity of three antioxidant enzymes, namely superoxide dismutase (SOD), catalase (CAT), and glutathione peroxidase (GPX), which are altered under various physiological states involving free radical attack [32]. The roles of mannitol as an osmo-protectant as well as free radical scavenger that influences the activities of antioxidant enzymes including SOD, CAT, glutathione reductase (GR), peroxidase (POX), and ascorbate peroxidase (APX) have also been established [33]. Previously, Iacopini et al. described catechin, epicatechin, and quercetin as phenolic compounds that can independently or synergistically exhibit DPPH radical activities [34]. However, in the present study, eriodictyol and caffeic acid were linked to tyrosinase inhibition and total flavonoid content assays, respectively. Notably, eriodictyol is a flavonoid that can inhibit melanogenesis [35].

## Conclusion

The present study construed the chemometric profiles of 51 plant samples across phylogenic groups and spatial parts, correlating their untargeted metabolite profiles with corresponding bioactivity phenotypes. Notwithstanding the effects of varying harvest time and regions, we observed distinct metabolomic profiles with higher antioxidant and tyrosinase inhibitory activities for leaf and stem extracts, respectively, across different families. Correlation analyses indicated that several metabolites either independently or synergistically affected the antioxidant phenotypes in plant extracts. Considering the subtle mechanisms, the observed chemical diversity for varying plant samples provided an insight of the specialized metabolic pathways which may have affected the phenotypic variance. Further, the holistic metabolite repertoire and associated bioactivities reported in the study can supplement the existing compendium of plant metabolomics data.

## Methods

### Chemicals and reagents

HPLC-grade water, ethanol, methanol, and acetonitrile were purchased from Fisher Scientific (Pittsburgh, PA, USA). Formic acid, N-methyl-N-(trimethylsilyl) trifluoroacetamide (MSTFA), Methoxyamine hydrochloride, pyridine, 2,2-diphenyl-1-pricrylhydrazyl (DPPH), sodium hydroxide, diethylene glycol, Folin-Ciocalteu’s phenol reagent, and sodium carbonate were purchased from Sigma Aldrich (St. Louis, MO, USA).

### Plant materials

We procure five different plant samples for each of the 51 species belonging to three different plant families from the National Institute of Biological Resources (NIBR, Incheon, Korea). The plant samples were dried under shade, pooled, and ground into fine homogeneous powders using the Mixer Mill. Information regarding the plants used in this study is listed in Table 1.

**Table 1:** Basic information about the 51 species of plant samples

No.	Species	Family	Collection date	Collection Site
1	*Hemistepta lyrata*	Asteraceae	2015-05-27	Hyeon-ri, Girin-myeon, Inje-gun, Gangwon-do
2	*Breea segeta*	Asteraceae	2015-06-08	Oksan-ri, Hojeo-myeon, Wonju-si, Gangwon-do
3	*Aster incisus*	Asteraceae	2015-08-11	Myeong-wol-ri, Sanae-myeon, Hwacheon-gun, Gangwon-do
4	*Syneilesis palmata*	Asteraceae	2015-08-13	Hantan-ri, Mitan-myeon, Pyeongchang-gun, Gangwon-do
5	*Sigesbeckia glabrescens*	Asteraceae	2015-08-29	Udu-ri, Dolsan-eup, Yeosu-si, Jeollanam-do
6	*Artemisia montana*	Asteraceae	2015-09-02	Namseo-ri, Seo-myeon, Ulleung-gun, Gyeongsangbuk-do
7	*Sigesbeckia pubescens*	Asteraceae	2015-09-04	Gujeol-ri, Yeoryang-myeon, Jeongseon-gun, Gangwon-do
8	*Synurus deltoides*	Asteraceae	2015-09-05	Hyeol-dong, Taebaek-si, Gangwon-do
9	*Cirsium japonicum*	Asteraceae	2015-09-07	Daepo-dong, Seogwipo-si, Jeju special self-governing province
10	*Sonchus asper*	Asteraceae	2015-09-08	Bomok-dong, Seogwipo-si, Jeju special self-governing province
11	*Atractylode ovata*	Asteraceae	2015-09-12	Baegil-ri, Gwayeok-myeon, Goheung-gun, Jeollanam-do
12	*Dendranthema boreale*	Asteraceae	2015-10-06	Sasong-ri, Baekgok-myeon, Jincheon-gun, Chungcheongbuk-do
13	*Helianthus tuberosus*	Asteraceae	2014-08-12	Jiro-ri, Byeongyeong-myeon, Gangjin-gun, Jeollanam-do
14	*Conyza canadensis*	Asteraceae	2014-08-14	Sangdodae-ri, Sangchon-myeon, Yeongdong-gun, Chungcheongbuk-do
15	*Artemisia capillaris*	Asteraceae	2014-08-21	Nadae-ri, Yaro-myeon, Hapcheon-gun, Gyeongsangnam-do
16	*Saussurea pulchella*	Asteraceae	2014-08-30	Gohan-ri, Gohan-eup, Jeongseon-gun, Gangwon-do
17	*Erigeron annuus*	Asteraceae	2014-08-05	Dongmak-ri, Yeoncheon-eup, Yeoncheon-gun, Gyeonggi-do
18	*Bidens bipinnata*	Asteraceae	2014-08-19	Dongmak-ri, Yeoncheon-eup, Yeoncheon-gun, Gyeonggi-do
19	*Lactuca indica*	Asteraceae	2014-08-24	Gomo-ri, Soheul-eup, Pocheon-si, Gyeonggi-do
20	*Vicia amoena*	Fabaceae	2015-08-11	Guun-ri, Sangseo-myeon, Hwacheon-gun, Gangwon-do
21	*Chamaecrista nomame*	Fabaceae	2015-08-18	Masan-dong, Gimpo-si, Gyeonggi-do
22	*Robinia pseudoacacia*	Fabaceae	2014-10-23	Sin-ri, Goryeong-eup, Goryeong-gun, Gyeongsangbuk-do
23	*Albizia julibrissin*	Fabaceae	2014-08-06	Daechi-ri, Daechi-myeon, Cheongyang-gun, Chungcheongnam-do
24	*Sophora flavescens*	Fabaceae	2014-08-18	Hanggok-ri, Gunbuk-myeon, Okcheon-gun, Chungcheongbuk-do
25	*Lespedeza cuneata*	Fabaceae	2014-08-22	Geogi-ri, Jusang-myeon, Geochang-gun, Gyeongsangnam-do
26	*Desmodium caudatum*	Fabaceae	2014-08-24	Seonheul-ri, Jocheon-eup, Jeju-si, Jeju special self-governing province
27	*Pueraria lobata*	Fabaceae	2014-08-04	Mamyeong-ri, Naechon-myeon, Pocheon-si, Gyeonggi-do
28	*Lespedeza maximowiczii*	Fabaceae	2011-08-03	Giri, Gajo-myeon, Geochang-gun, Gyeongsangnam-do
29	*Lespedeza bicolor*	Fabaceae	2011-08-04	Ongdong-myen, Jeongeup-si, Jeollabuk-do
30	*Lespedeza cyrtobotrya*	Fabaceae	2011-07-02	Wang-dong, Gwangsan-gu, Gwanju
31	*Rubus corchorifolius*	Rosaceae	2015-04-22	Cheongyong-ri, Gogeum-myeon, Wando-gun, Jeollanam-do
32	*Spiraea prunifolia*	Rosaceae	2015-04-29	Jindong-ri, Girin-myeon, Inje-gun, Gangwon-do
33	*Rubus parvifolius*	Rosaceae	2015-06-02	Gwansan-ri, Yaksan-myeon, Wando-gun, Jeollanam-do
34	*Rosa multiflora*	Rosaceae	2015-06-03	Gwansan-ri, Yaksan-myeon, Wando-gun, Jeollanam-do
35	*Prunus sargentii*	Rosaceae	2015-06-08	Oe-ri, Yeongheung-myeon, Ongjin-gun, Incheon
36	*Prunus davidiana*	Rosaceae	2015-06-30	Sanggeol-ri, Dong-myeon, Chuncheon-si, Gangwon-do
37	*Sanguisorba tenuifolia*	Rosaceae	2015-09-17	Donggo-ri, Sinji-myeon, Wando-gun, Jeollanam-do
38	*Sorbus commixta*	Rosaceae	2014-07-16	Jeodong-ri, Ulleung-eup, Ulleung-gun, Gyeongsangbuk-do
39	*Prunus armeniaca*	Rosaceae	2014-07-20	Ojeong-dong, Daedeok-gu, Daejeon
40	*Pyrus ussuriensis*	Rosaceae	2014-08-01	Icheon-ri, Sangbuk-myeon, Ulju-gun, Ulsan
41	*Prunus yedoensis*	Rosaceae	2014-08-07	Janghyeon-ri, Cheongna-myeon, Boryeong-si, Chungcheongnam-do
42	*Spiraea salicifolia*	Rosaceae	2014-08-08	Ungyo-ri, Bangnim-myeon, Pyeongchang-gun, Gangwon-do
43	*Chaenomeles sinensis*	Rosaceae	2014-08-10	Ojeong-dong, Daedeok-gu, Daejeon
44	*Eriobotrya japonica*	Rosaceae	2014-08-13	Jiro-ri, Byeongyeong-myeon, Gangjin-gun, Jeollanam-do
45	*Rubus coreanus*	Rosaceae	2014-08-14	Sogye-ri, Hwanggan-myeon, Yeongdong-gun, Chungcheongbuk-do
46	*Rubus crataegifolius*	Rosaceae	2014-08-21	Nadae-ri, Yaro-myeon, Hapcheon-gun, Gyeongsangnam-do
47	*Rubus phoenicolasius*	Rosaceae	2014-08-21	Nadae-ri, Yaro-myeon, Hapcheon-gun, Gyeongsangnam-do
48	*Pourthiaea villosa*	Rosaceae	2014-08-24	Seonheul-ri, Jocheon-eup, Jeju-si, Jeju special self-governing province
49	*Prunus maackii*	Rosaceae	2014-08-30	Gurae-ri, Sangdong-eup, Yeongwol-gun, Gangwon-do
50	*Prunus padus*	Rosaceae	2014-05-22	Gohan-ri, Gohan-eup, Jeongseon-gun, Gangwon-do
51	*Prunus sp.*	Rosaceae	2014-08-08	Gomo-ri, Soheul-eup, Pocheon-si, Gyeonggi-do

### Sample extract preparation

Approximately 1 g of sample powder was extracted using 10 mL of 80% methanol following continuous shaking at 200 rpm for 24 h. The resulting mixture was cold centrifuged (4 °C) at 2800×*g* for 15 min (Hettich Zentrifugen, Universal 320), and the supernatant was filtered using a 0.2 μm syringe. The supernatant was dried under a speed-vacuum concentrator (Modulspin 31, Biotron, Korea), and was resuspended in 80% methanol at an appropriate concentration. This suspension was then analyzed by UHPLC-Q-Orbitrap-MS to detect secondary metabolites. For GC-TOF-MS analysis, extracts were oximated using methoxyamine hydrochloride (20 mg mL^− 1^) in pyridine at 30 °C for 90 min. Then, the oximated samples were silylated with MSTFA at 37 °C for 30 min. All MS analyses were conducted with three analytical replications.

### Bioactivity assays

#### DPPH assay

Measurement of antioxidant activity was carried out with DPPH assays, following the methodology originally proposed by Villano et al. [36], with some modifications. In brief, the DPPH (200 μmol) reagent was dissolved in ethanol and maintained for 20 min at 60–70 °C until the solution absorbance reached 1.0 ± 0.02 at 515 nm, as measured by a spectrophotometer (Thermo Electron, Spectronic Genesys 6, Madison, WI, USA). The resulting solution was kept stable for the next 16 h and stored at 4 °C. The assays were performed by adding 180 μL of the DPPH solution to the plant sample extracts (20 μL, 1 mg mL^− 1^), and the resulting mixture was incubated for 20 min at 37 °C in the dark. The reaction absorbance was measured at 515 nm. Results were expressed as Trolox equivalent activity concentrations (mM), and as the mean value of the three analytical replicates.

#### Total phenol content

Total phenol content assays were performed in two steps. First, the reaction mixture, containing 20 μL of plant sample extract in 80% methanol (1 mg mL^− 1^) and 100 μL of 0.2 N Folin-Ciocalteu’s phenol reagent, was incubated for 5 min in the dark. Then, 80 μL of 7.5% Na_2_CO^3^ was added, and the resulting reaction mixture was incubated for 60 min. Finally, absorbance was measured at 750 nm. Assay results were expressed in terms of gallic acid equivalent of the activity (μg mL^− 1^), and as the mean value of three analytical replicates.

#### Total flavonoid content (stock 1000 ppm)

For total flavonoid content assays, reaction mixtures contained 20 μL of plant sample extract in 80% methanol (1 mg mL^− 1^), 20 μL of 0.1 N NaOH, and 160 μL of 90% diethylene glycol. The reaction mixture was incubated for 60 min and the resulting absorbance was recorded at 405 nm. Results were expressed as naringin equivalent activity concentrations (μg mL^− 1^). The data were presented as the mean of three analytical replicates.

#### Tyrosinase inhibitory activity

Mushroom tyrosinase inhibitory activity was determined using the following method. A reaction mixture was prepared with 125 μL of 0.1 M sodium phosphate buffer (pH 6.5), 5 μL of plant sample extract in 80% methanol (10 mg mL^− 1^), 30 μL of mushroom tyrosinase (1000 unit mL^− 1^), and 40 μL of 1.5 mM L-tyrosine, and was added to 96-well plates. The reaction mixture was incubated at 37 °C for 20 min and absorbance was measured at 490 nm. The data were presented as the mean value of three analytical replicates.

### Mass spectrometry (MS) analysis

#### GC-TOF-MS analysis

An Agilent 7890A gas chromatography (GC) system equipped with an Agilent 7693 autosampler coupled to a Pegasus Time-of-Flight Mass Spectrometer (TOF-MS) detector (Leco Corporation, St. Joseph, MI, USA) was used for GC-TOF-MS analyses as described by Lee et al. [13].

#### UHPLC-Q-Orbitrap-MS analysis

Samples were analyzed using a Q-Exactive Orbitrap MS equipped with a heated electrospray ionization source (Thermo Fischer Scientific, CA, USA), which consisted of a DIONEX UltiMate 3000 UHPLC system (Ultimate 3000 RS pump, Ultimate 3000 RS column compartment, and Ultimate 3000 RS autosampler; Dionex Corporation, CA, USA). Samples were separated on a hypersil gold C18 selectivity LC column (i.d., 1.9 μm, 50 × 2.1 mm; Thermo Fisher scientific) at a column oven temperature of 25 °C. The mobile phases consisted of 0.1% formic acid in water (B) and in acetonitrile (C), and the compositions of the gradient flows were the same. The gradient was gradually increased from 0% solvent C to 100% solvent C over 20 min, and was maintained for a further 2 min. The flow rate was 0.3 mL min^− 1^ and the injection volume was 10 μL. Mass spectra were obtained using electrospray ionization in negative and full scan modes within a range of m/z 100–1000. The operating parameters were as follows: spray needle voltage, ± 3.3 kV; capillary temperature, 320 °C; probe heater temperature, 300 °C; stacked ring ion guide (S-lens) radio frequency (RF) level, 60%; resolution (full-width at half-maximum; FWHM), 35,000.

#### Ultra performance liquid chromatography-quadrupole-time of flight mass spectrometry (UPLC-Q-TOF-MS) analysis

UPLC-Q-TOF-MS analyses were performed using a Waters Micromass Q-TOF Premier as described by Son et al. [15]. The mobile phase consisted of 0.1% v/v formic acid in water (A) and in acetonitrile (B). The solvent gradient system consisted of the following: B was increased from 5 to 100% (v/v) over 11 min and was maintained at 100% for 12 min. Then, B was decreased to 5% in 0.01 min, and was maintained at this level up to 13 min. The sample injection volume was 5 μL and the flow rate was maintained at 0.3 mL min^− 1^.

### Data processing and multivariate analysis

GC-TOF-MS data files were converted to CDF format using ChromaTOF software v4.44 (Leco Co., CA, USA). LC-MS data (*.raw) were converted to netCDF (*.cdf) format using Xcalibur (version 2.2; Thermo Fischer Scientific, CA, USA). After conversion, the CDF format data were processed using the metAlign software package, and SIMCA-P + 12.0 (Umetrics, Umea, Sweden) for the principal component analysis (PCA) and partial least squares discriminant analysis (PLS-DA) modeling as described by Lee et al. [13]. The significantly different (*p* value < 0.05) metabolites contributing to the statistical variance among plant species were tested using one-way ANOVA on STATISCA (version 7.0, StaSoft Inc., Tulsa, OK, USA).

In the antioxidant and tyrosinase inhibition activity tests, differences were discerned by t-tests using PASW Statistics 18 (SPSS Inc., Chicago, IL, USA). Pairwise correlations between metabolites and bioactivities (antioxidant activity and tyrosinase inhibition activity) were calculated by Pearson’s correlation coefficient using PASW Statistics 18. The correlations between metabolites and antioxidant bioactivity were visualized using heat map representations made with MEV software 4.8 (multiple array viewer, http://www.tm4.org/).

## Supplementary information

**Additional file 1.** Tentatively identified primary metabolits in plants based on metabolite profiling with multivariate analysis.

**Additional file 2.** Tentatively identified secondary metabolits in plants based on metabolite profiling with multivariate analysis.

## Data Availability

The data sets supporting the results of this article are included within the article and its additional files.

## Abbreviations

AAA: Aromatic amino acid
APX: Ascorbate peroxidase
BCAA: Branch chain amino acid
CAT: Catalase
DPPH: 2,2-diphenyl-1-pricrylhydrazyl
GC-TOF-MS: Gas chromatography time-of-flight mass spectrometry
GPX: Glutathione peroxidase
GR: Glutathione reductase
HPLC: High-performance liquid chromatography
MS: Mass spectrometry
MSTFA: Methoxyamine hydrochloride, N-methyl-N-(trimethylsilyl) trifluoroacetamide
PCA: Principal component analysis
PLS-DA: Partial least squares discriminant analysis
POX: Peroxidase
SOD: Superoxide dismutase
UHPLC-Q-orbitrap-MS/MS: Ultrahigh-performance liquid chromatography quadrupole orbitrap ion trap tandem mass spectrometry
VIP: Variable importance in the projection
